# Age-associated trajectories of body composition at high altitude

**DOI:** 10.1016/j.jnha.2026.101005

**Published:** 2026-10-09

**Authors:** Martin Burtscher, Hannes Gatterer, Johannes Burtscher

**Affiliations:** a Department of Sport Science, University of Innsbruck, 6020, Innsbruck, Austria; b Institute of Mountain Emergency Medicine, Eurac Research, 39100, Bolzano, Italy; c Department of Psychiatry, Psychotherapy, Psychosomatics and Medical Psychology, University Hospital for Psychiatry II, Medical University of Innsbruck, 6020, Innsbruck, Austria

**Keywords:** Altitude, Aging, Sex, Muscle, Fat

Body composition is an important determinant of health and varies substantially both within and between populations. The geographic patterning of these differences likely reflects a complex interplay of ancestry and genetic background, environmental conditions such as temperature and altitude, behavioral factors including diet and physical activity, and social and historical determinants such as migration, socioeconomic conditions, conflict, and colonization. However, the relative contribution of these factors remains incompletely understood, and their interactions make it difficult to disentangle their individual effects [1,2]. Despite this substantial heterogeneity, aging is generally associated with progressive changes in body composition, including declines in skeletal muscle and fat-free mass and alterations in adipose tissue distribution, with trajectories that differ between men and women [3]. However, most evidence on age- and sex-related trajectories of body composition comes from populations living at low altitude, and it remains unclear whether chronic exposure to the lower oxygen availability (hypoxia) at higher elevations modifies these trajectories. This question is particularly relevant for high-altitude populations, where chronic exposure to hypoxia may influence appetite and food intake, energy expenditure, muscle metabolism, fluid homeostasis, and behavior. Against this background, a recent study of 1,318 native Tibetan adults of both sexes, aged 20–85 years, provides important evidence on the interplay between aging and chronic altitude exposure [4]. By including participants living at three different altitudes (2,800, 3,650, and 4,500 m), the study allowed the investigators to assess nonlinear age-related patterns in body composition and their associations with sex and altitude within a cross-sectional design [4]. The authors identified altitude-specific turning points, defined as the estimated ages at which the direction of the association between age and body composition components, such as appendicular skeletal muscle mass index (ASMI) and fat mass index (FMI), changes [4].

Yang et al. demonstrated that age-related body-composition trajectories among native Tibetans living at 2,800–4,500 m broadly resemble those described at low altitude [3,5], with ASMI increasing during early adulthood before declining after approximately 43 years in men and 49 years in women [4]. In contrast, the age-related trajectory of adiposity appeared to be modified by altitude, particularly in women, in whom the FMI turning point occurred substantially earlier at 4,500 m (50 years) than in the overall cohort (69 years) [4] (Fig. 1). These findings suggest that the level of chronic hypoxia may influence the timing of age-related changes in body composition, although longitudinal studies are required to distinguish altitude-related effects from cross-sectional, lifestyle, nutritional, or population-specific differences.

**Fig. 1:**
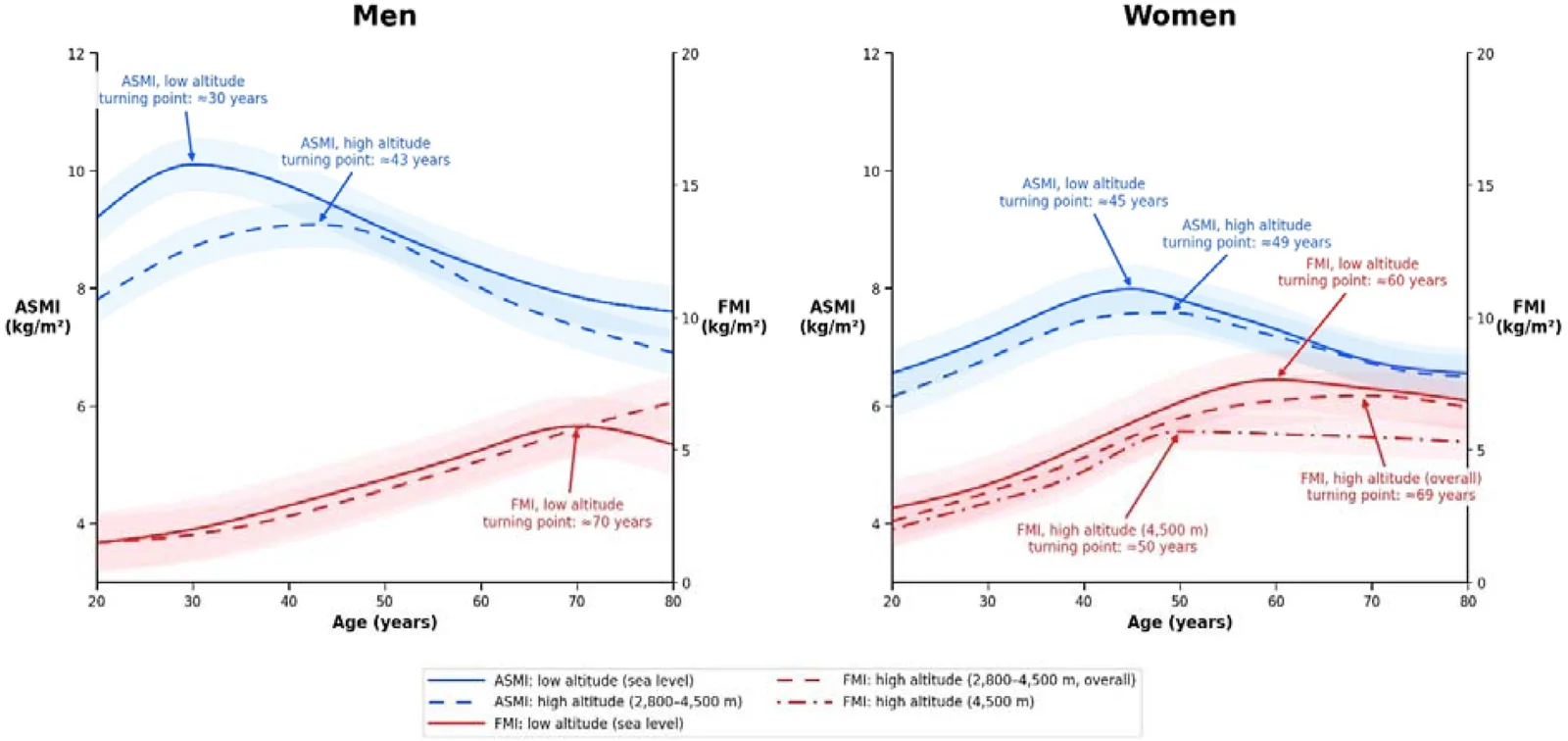
Schematic comparison of age- and sex-associated appendicular skeletal muscle mass index (ASMI) and fat mass index (FMI) trajectories between low and high altitude (based on data from Refs. [4,6]).

The findings of Yang et al. complement previous research in Tibetan populations. For example, Ye et al. studied 2,318 Tibetan adults and found that muscle mass and functional measures relevant to sarcopenia differed from conventional lowland reference values, with a substantial prevalence of sarcopenia among those older than 60 years [7]. They proposed population-specific reference values based on skeletal muscle mass, grip strength, and gait speed. Yang et al. extend these findings by addressing not the thresholds defining sarcopenia, but the age-related trajectory of skeletal muscle mass across adulthood [4]. Although cross-sectional data require cautious interpretation, the observed ASMI turning points at approximately 43 years in men and 49 years in women raise the possibility that, beyond differences in population-specific reference values, the age- and sex-related trajectories of skeletal muscle mass may also vary in high-altitude populations. Accordingly, altitude-specific age-related trajectories could complement conventional sarcopenia reference values and potentially facilitate earlier identification of individuals at increased risk of muscle loss. Given the established associations of muscle mass and sarcopenia with cardiometabolic health and mortality, such trajectories may also contribute to a broader assessment of health risk in high-altitude populations [8,9].

As acknowledged by the authors, their findings may remain subject to residual confounding despite adjustment for lifestyle factors, including the Physical Activity Index and Diet Quality Index, because other potentially relevant variables, such as socioeconomic status, access to healthcare, and environmental factors, were not accounted for. For example, lower ambient temperatures, which decrease by approximately 6.5 °C per 1,000 m increase in altitude under standard atmospheric conditions, may increase energy expenditure and influence adipose-tissue metabolism through coordinated thermoregulatory adaptations that help maintain core temperature [10]. These metabolic responses may be accompanied by compensatory changes in energy intake and behavior, including physical activity. The cross-sectional design, however, limits causal and individual-level interpretation of the observed age-related associations and estimated turning points, which should not be regarded as definitive ages at which changes in muscle or fat mass begin. Cohort effects, selective survival, and unmeasured altitude-related factors may also have contributed to the observed patterns. Longitudinal studies are therefore needed to establish individual trajectories and determine whether altitude independently modifies age-related changes in body composition.

Overall, the findings presented by Yang et al. highlight the potential importance of considering altitude as an environmental factor when interpreting age- and sex-related differences in body composition and their implications for health.

## Authors contribution

Conceptualization and first draft: MB; Writing-review & editing: MB, HG, JB; all authors approved the final version of the manuscript.

## Ethics declaration

The authors declare that the study does not involve humans nor animal subjects.

## Declaration of Generative AI and AI-assisted technologies in the writing process

DeepL and ChatGPT were used solely to assist with language editing and refinement and to support the preparation of the schematic figure. All information and AI-assisted output were critically reviewed and carefully verified by the authors, who remain fully responsible for the accuracy and integrity of the final manuscript.

## Funding

No specific funding was received for this work.

## Declaration of competing interest

None of the authors declared any conflicts of interest in connection with this manuscript.
